# Estimated Nucleotide Reconstruction Quality Symbols of Basecalling Tools for Oxford Nanopore Sequencing

**DOI:** 10.3390/s23156787

**Published:** 2023-07-29

**Authors:** Wiktor Kuśmirek

**Affiliations:** Institute of Computer Science, Warsaw University of Technology, 00-661 Warsaw, Poland; wiktor.kusmirek@pw.edu.pl

**Keywords:** basecalling, FASTQ, quality evaluation, nanopore, bioinformatics

## Abstract

Currently, one of the fastest-growing DNA sequencing technologies is nanopore sequencing. One of the key stages involved in processing sequencer data is the basecalling process, where the input sequence of currents measured on the nanopores of the sequencer reproduces the DNA sequences, called DNA reads. Many of the applications dedicated to basecalling, together with the DNA sequence, provide the estimated quality of the reconstruction of a given nucleotide (quality symbols are contained on every fourth line of the FASTQ file; each nucleotide in the FASTQ file corresponds to exactly one estimated nucleotide reconstruction quality symbol). Herein, we compare the estimated nucleotide reconstruction quality symbols (signs from every fourth line of the FASTQ file) reported by other basecallers. The conducted experiments consisted of basecalling the same raw datasets from the nanopore device by other basecallers and comparing the provided quality symbols, denoting the estimated quality of the nucleotide reconstruction. The results show that the estimated quality reported by different basecallers may vary, depending on the tool used, particularly in terms of range and distribution. Moreover, we mapped basecalled DNA reads to reference genomes and calculated matched and mismatched rates for groups of nucleotides with the same quality symbol. Finally, the presented paper shows that the estimated nucleotide reconstruction quality reported in the basecalling process is not used in any investigated tool for processing nanopore DNA reads.

## 1. Introduction

DNA sequencing is one of the fastest-growing fields of modern science [1]. Over the years, many sequencing technologies have been created; currently, one of the most popular is nanopore sequencing. The nanopore reads are used in research in a range of scientific fields, inter alia, genome assembly, and detecting structural DNA variants [2,3,4,5,6].

Briefly, nanopore sequencing depends on threading a single strand of DNA through a nanoscale hole, called the nanopore [7]. As the DNA strand flows through the nanopore, the changes in the ionic current caused by differences in the shifting nucleotide sequences occupying the nanopore are detected [8]. Then, the changes in the ionic current value are segmented as discrete events, creating a series of discrete values of the detected current on the specified nanopore [9]. Finally, the basecalling stage consists of converting a set of discrete ionic current values into DNA reads.

There are many applications for basecalling process using different algorithms to reconstruct nucleotides. The oldest and most basic algorithm for reconstructing nucleotides from current values is the hidden Markov model (HMM) based on Viterbi-decoding algorithms. In this group of methods, HMM is used to convert current measurements to events, and the Viterbi algorithm is used to decode the nucleotides. The described procedures are implemented in Metrichor and Nanocall [10] tools. Nowadays, the most popular methods for nucleotide reconstruction are algorithms based on artificial intelligence. The techniques adapted to the basecalling process include recurrent neural network RNN (DeepNano [11], basecRAWller [12], Nanonet), the bidirectional WaveNets [13] (WaveNano [14]), convolutional neural network CNN (Causalcall [15], Chiron [16]) and connectionist temporal classification (CTC) [17] (Causalcall [15], Chiron [16]).

The basecalling application output is a FASTA or FASTQ file. The FASTQ format is an extension of the FASTA format containing information about the quality of reconstruction of each nucleotide. Unfortunately, more than half of basecallers do not provide information about the estimated quality of the reconstructed nucleotide. For example, the Nanocall [10] tool authors explain the lack of information about the estimated quality of reconstruction by the fact that the individual base error rate is too high for base qualities to be informative. However, some of the basecallers provide information about each nucleotide’s estimated reconstruction quality.

As there are many applications for basecalling, there are several different ways to estimate the quality of a reconstructed nucleotide. Firstly, the Chiron and Causalcall tools calculate the quality score as follows: $qs=10\times \log_{10}\left(P1/P2\right)$, where qs is the quality score, P1 is the probability of occurrence of the most likely nucleotide, and P2 is the probability of occurrence of the second likely nucleotide. Secondly, the Flappie and the Nanonet tools derive reconstruction quality scores directly from the trained model. Both applications calculate quality as follows: $qs=-10\cdot (y\cdot \log_{10}\left(1-p\right)+\log_{10}\left(x\right))$, where qs is the quality score, *p* is the probability of occurrence of the most likely nucleotide, *x* and *y* are the internal application variables that scale the quality value (Table 1). Finally, for the Albacore and Guppy tools, the method of estimating the reconstruction quality score is not published, the basecallers are closed-source, which limits our insights into their quality estimation formula.

So far, implemented basecallers have been compared in multiple studies. In [18], the authors compared basecallers in terms of the identity of the reconstructed DNA sequence, including the reads after the basecalling process and the consensus built from them.

The study also presented changes in individual basecallers over subsequent versions of the software, the impact of polishing DNA sequences, and the division of errors in the resultant DNA sequences from multiple categories, like substitution, deletion, insertion, etc. The studies that describe new basecallers compared the tools in other criteria, like calculation time [10,11,12,14,15,16], the quality of DNA reads (based on mapping the reads to the reference genome) [10,11,12,14,15,16], the quality of the de novo assembling process [15,16], the prevalence of repetitive 6 mers [11].

Unfortunately, none of the articles addressed the problem of the correctness and usefulness of information about the nucleotide’s estimated reconstruction quality. Herein, we compare several basecallers in terms of the estimated quality of the predicted single nucleotide (fourth line of the FASTQ file). We also examine how the nucleotide’s estimated reconstruction quality affects further stages of processing long nanopore DNA reads.

## 2. Materials and Methods

The first step of the research methodology presented involves masking repetitive regions in the reference genome. The DNA of many organisms contains repetitive fragments, i.e., repeats of the same (or very similar motif) in many regions of the genome [19]. The presence of repetitive fragments in DNA negatively affects the quality of many different genetic data processing processes, e.g., de novo assembling [20] or mapping DNA reads to a reference genome [21]. To overcome this issue, we first identify repetitive sequences using the RepeatModeler [22] tool. Then, RepeatMasker [23] software is applied to change all of the repetitive nucleotides to ‘N’ signs.

The second step of the presented workflow involves mapping long DNA reads to the masked reference genome via the minimap2 [24] tool. The mentioned mapping is conducted with the parameter map-ont, and the mapping results are stored in the SAM file.

The last stage of the research methodology presented is to calculate statistics based on the number of correctly mapped nucleotides and the number of mapping errors. Here, we calculate several parameters, like the number of matched (the same nucleotide in the DNA read and reference genome), mismatched (the single nucleotide that is different in the DNA read and reference genome), improperly inserted (the nucleotide present in the DNA read and not present in the reference genome), and clipped nucleotides (nucleotides not mapped at both ends of the alignment). The calculated statistics are divided into groups based on the nucleotide’s estimated reconstruction quality sign from the FASTQ file; all mentioned parameters are calculated based on the CIGAR string reported by the minimap2 mapping tool in the SAM file.

## 3. Results

### 3.1. Basecaller Comparison

The experiments were conducted on three publicly available datasets on *Acinetobacter pittii*, *Haemophilus haemolyticus*, and *Serratia marcescens* genomes, sequenced using MinION Flow Cell (R9.4.1) (Oxford Nanopore Technologies, Oxford, United Kingdom). The datasets were downloaded as raw fast5 files from the [18] study, composed of 4467, 8670, and 16,743 files for *Acinetobacter pittii*, *Haemophilus haemolyticus*, and *Serratia marcescens*, respectively. The downloaded data were basecalled by Albacore (ver. 3.0.1), Causalcall (commit f5ab3db), Chiron (ver. 0.6.1.1), Dorado [25] (ver. 0.2.4), Flappie (ver. 2.1.3), Guppy (ver. 3.3.0), and Nanonet (ver. 2.0.0). The launch parameters for all applications are posted in the publicly available repository: https://github.com/wkusmirek/basecalling-quality (accessed on 13 June 2023). To explore the effect of the training set on the estimation reconstruction quality performance, for the Dorado basecaller, we used three trained, default models (dna_r9.4.1_e8_fast@v3.4, dna_r9.4.1_e8_hac@v3.3, and dna_r9.4.1_e8_sup@v3.3), and for the Guppy tool, we used the default model and two custom trained models downloaded from the [18] study (the Guppy tools with custom models are marked in the presented paper as ‘Guppy kp’ and ‘Guppy kp-big-net’). Briefly, the models were trained by the Sloika neural network training toolkit (https://github.com/nanoporetech/sloika (accessed on 13 June 2023)) on the training dataset composed of reads from 50 different isolate genomes. The final training dataset contained 226,166 DNA reads for training the neural network and 1000 DNA reads for validating the results. A detailed description of the model’s training process and the scripts used can be found in reference [18].

As a result of the basecalling process, we obtained 33 sets of long DNA reads; the basic statistics of the obtained reads calculated with the BBMap [26] tool are presented in Table 2. Firstly, the number of reads basecalled by the Albacore, Causalcall, Chiron, Dorado, Flappie, and Guppy tools were equal to the number of raw fast5 files obtained from the nanopore sequencer. However, the Nanonet tool produced almost twice as many DNA sequences: 7702 reads from 4467 raw fast5 files (*Acinetobacter pittii*), 15,956 reads from 8669 raw fast5 files (*Haemophilus haemolyticus*), and 34,046 reads from 16,742 raw fast5 files (*Serratia marcescens*). Moreover, the sum of DNA reads basecalled by the Nanonet tool was approximately equal to the sum of sequence lengths produced by the other tools, e.g., Albacore. The average length of the basecalled DNA reads we obtained from the Nanonet tool was approximately half the average length of the DNA reads from the other tools, like Albacore. The poor performance of the Nanonet application was also demonstrated in [18], which confirms that the tool should not be selected for target basecalling in real nanopore DNA sequencing projects. Secondly, the Causalcall, Chiron, and Nanonet tools basecalled DNA reads, which, depending on the dataset, achieved a different mapping factor to the reference genome. For example, the DNA reads basecalled by the Chiron tool were mapped in 81.88%, 43.05%, and 84.50% of the reference genomes for *Acinetobacter pittii*, *Haemophilus haemolyticus*, and *Serratia marcescens*, respectively. Thirdly, the basecalled DNA reads by the Causalcall, Chiron, and Nanonet tools, mapped to the reference genome, were characterized by lower matched rates than the Albacore, Flappie, and Guppy results. Moreover, the Dorado tool with the ‘sup’ model produced DNA reads with the fewest substitution errors among other basecallers for all three investigated datasets.

**Figure 1:**
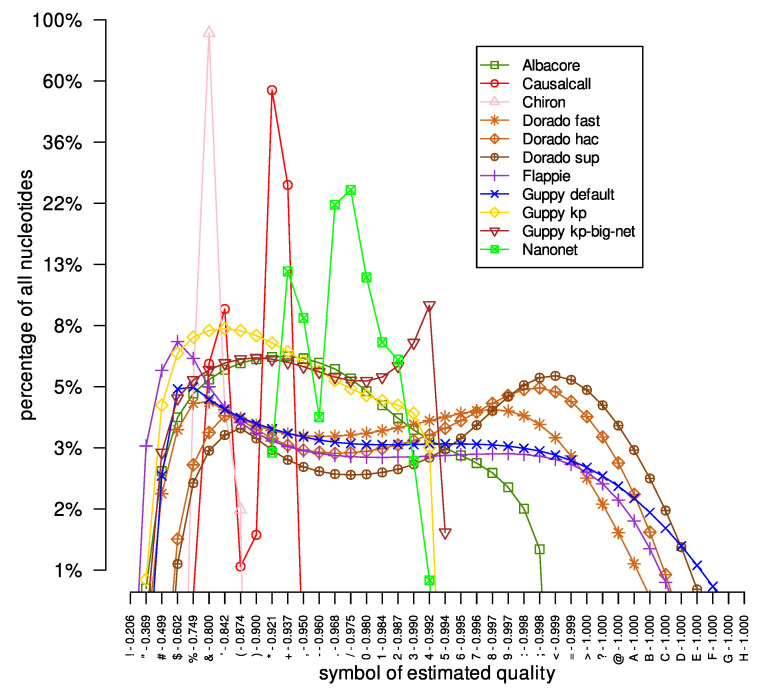
Reconstructed DNA reads of quality symbol distribution for the *Acinetobacter pittii* dataset. The x-axis represents successive symbols of reconstruction quality, the y-axis defines the content of nucleotides of the given reconstruction quality in the entire set of DNA reads. For example, Albacore provided a set of DNA reads in which approximately 3% of the recovered nucleotides were assigned a quality symbol of ‘5’, which theoretically signifies the probability of correct reconstruction at the 0.994 level. For the y-axis, a base-10 log scale was used.

We also checked the distribution of the estimated quality symbols in the investigated DNA reads datasets obtained by different basecallers. The results are presented in Figure 1. Firstly, different basecallers provide estimated quality symbols from different ranges. For example, the symbols provided by the Flappie application were in the range of ‘"’ to ‘B’, while the Nanonet tool basecalled DNA sequences with quality signs between ‘*’ and ‘3’. Moreover, the same tool with different models outputted nucleotides with estimated quality symbols of a different range. For example, the Guppy tool provides nucleotides with quality symbols ‘#’-‘E’, ‘#’-‘4’, and ‘#’-‘5’ for ‘default’, ‘kp’, and ‘kp-big-net’ models, respectively. Moreover, a different range of quality symbols affects the maximum number of a given quality symbol. For example, for the Albacore tool, the most common quality symbol (‘*’) represents 7% of all of Albacore’s quality symbols. On the other hand, the quality sign ‘&’ represents about 90% of all quality symbols provided by the Chiron tool. The reason for such a large difference in the number of the most common quality symbols is the breadth of the range of quality symbols provided: 4 and 25 other quality signs in the Chiron and Albacore results, respectively. Secondly, the curves representing the distribution of quality symbols for the Causalcall, Chiron, and Nanonet tools are irregular, with large variations in the count values for successive quality symbols, e.g., changes in tens of percents. On the other hand, for the Flappie and Dorado tools, the curves are much smoother, without significant changes in abundance for successive quality symbols. Thirdly, the curves representing the distribution of quality symbols in the results provided by individual basecallers are very similar in all analyzed datasets (see Supplementary Materials, Figures S1 and S2). For example, the curve of the Flappie application grows rapidly at the beginning, reaching its peak around the ‘&’ and ‘%’ signs, before slowly descending (the same descent curve shape for all three datasets) to the ‘A’ and ‘B’ signs.

After analyzing basic statistics of basecalled DNA reads, we prepared reference genomes by masking repetitive regions in raw reference sequences downloaded from the [18] study. As a result of this process, we masked 89,222 bp out of 3,814,719 bp (2.34%), 13,860 bp out of 2,042,591 bp (0.68%), and 241,685 bp out of 5,517,578 bp (4.38%) for *Acinetobacter pittii*, *Haemophilus haemolyticus*, and *Serratia marcescens* reference genomes, respectively.

**Figure 2:**
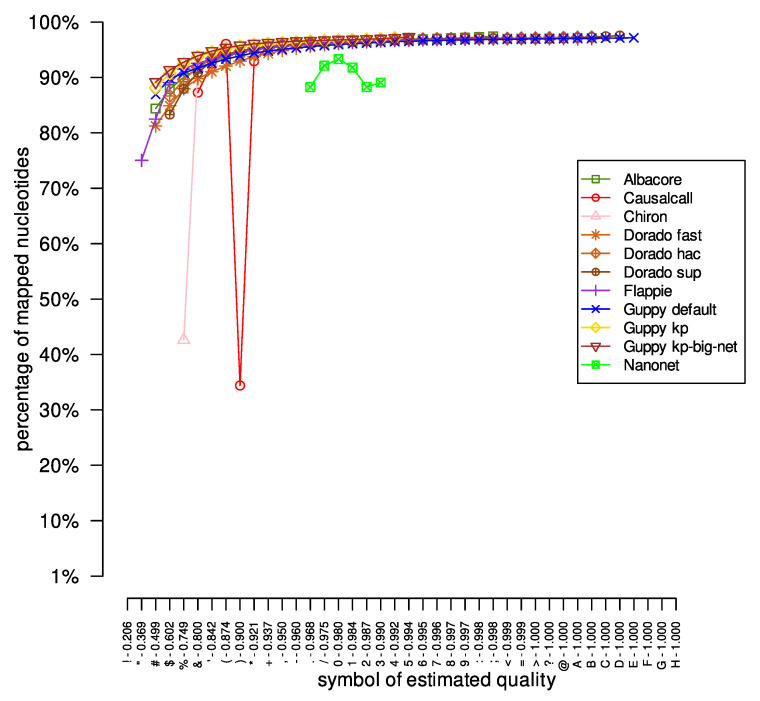
The number of nucleotides mapped by the minimap2 tool for the *Acinetobacter pittii* dataset. The ‘percentage of mapped nucleotides’ value was calculated as the quotient of the number of mapped nucleotides with a given symbol of the estimated quality and the number of all nucleotides with the same character of estimated quality.

Then, we mapped the long DNA reads onto the previously masked reference genomes. After mapping, for each symbol of estimated quality, we calculated the ratio of the number of mapped nucleotides (nucleotides reported in the SAM file) to the number of all nucleotides with the specified quality sign (nucleotides reported in the FASTQ file). The results are presented in Figure 2. As expected, with the successive symbols of estimated quality, the ratio of mapped to total nucleotides increases for the results obtained from the Albacore, Dorado, Flappie, and Guppy tools. However, for the DNA reads basecalled by the Causalcall, Chiron, and Nanonet tools, there are situations where even nucleotides with quality symbols denoting more certain probabilities of correct reconstruction were mapped to the reference genome in smaller numbers. Moreover, for the Nanonet application, there are quality symbols that appear in the DNA reads, but not in the mapping results (nucleotides with a given quality symbol do not map to the reference genome). For example, for the *Acinetobacter pittii* dataset, the Nanonet results in basecalled reads with estimated quality symbols from symbols ‘*’ to ‘3’. At the same time, the SAM file contains only nucleotides with an estimated reconstruction quality ranging from ‘.’ to ‘3’.

**Figure 3:**
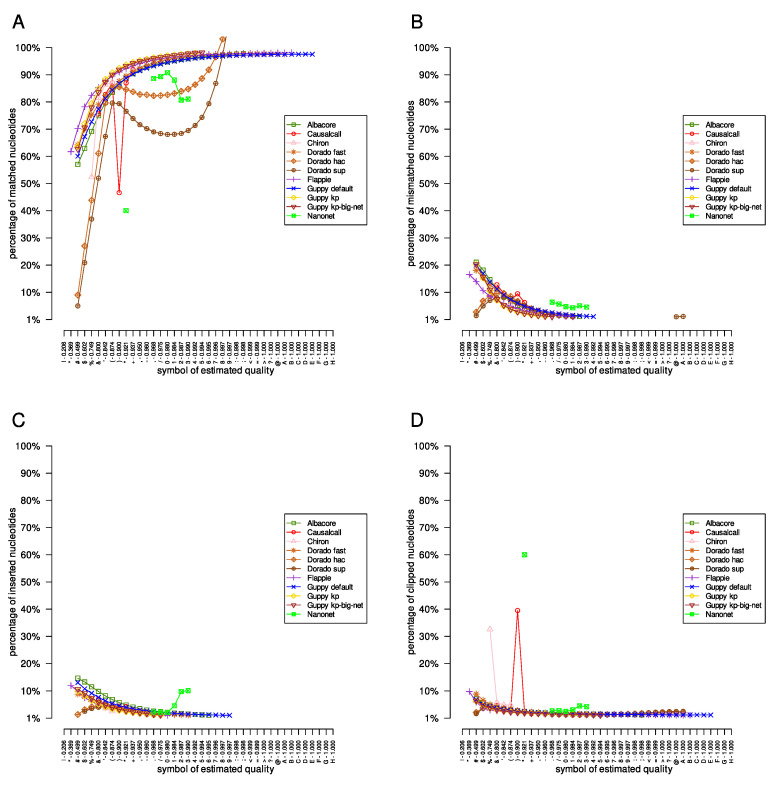
The results of the evaluation of the *Acinetobacter pittii* dataset. The nucleotides classified by the mapping processes as matched, mismatched, inserted, and clipped are presented in panels (**A**–**D**), respectively.

Finally, we computed statistics from each SAM file obtained. The results for the *Acinetobacter pittii* dataset are presented in Figure 3. Briefly, results obtained from the Albacore, Flappie, and Guppy applications had similar characteristics, as expected; the number of matched nucleotides increased with the successive symbols of the estimated quality, and the number of mismatched, inserted, and clipped nucleotides decreased. However, the performance characteristics of the Causalcall, Chiron, and Nanonet applications are irregular. For example, the number of improperly inserted nucleotides for the Nanonet tool results for all three investigated datasets increases with the successive symbols of quality. It is also worth following the course of the curves for the Dorado application; depending on the model used (‘fast’, ‘hac’, or ‘sup’), the curves are different.

### 3.2. R10.4 Flow Cell

**Figure 4:**
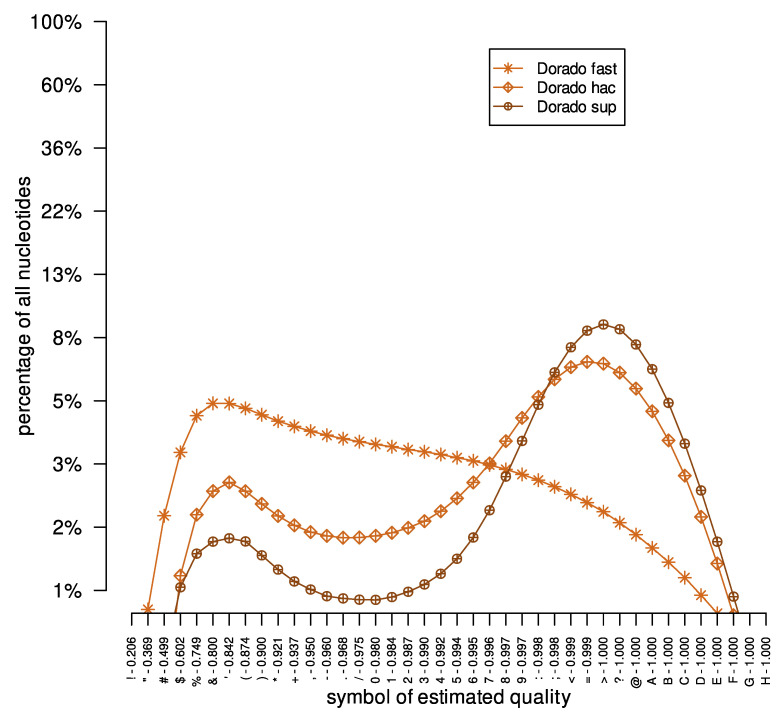
Reconstructed DNA reads of quality symbol distribution for the *Escherichia coli* dataset. The x-axis represents successive symbols of reconstruction quality, the y-axis defines the content of nucleotides of the given reconstruction quality in the entire set of DNA reads. For the y-axis, a base-10 log scale is used.

**Figure 5:**
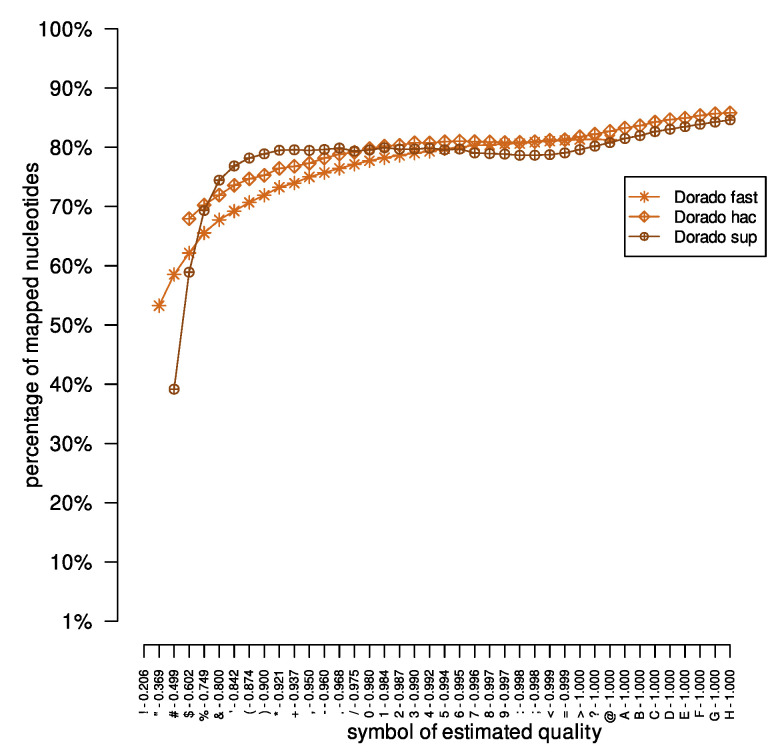
The number of nucleotides mapped by the minimap2 tool for the *Escherichia coli* dataset. The ‘percentage of mapped nucleotides’ value was calculated as the quotient of the number of mapped nucleotides with a given symbol of the estimated quality and the number of all nucleotides with the same character of estimated quality.

**Figure 6:**
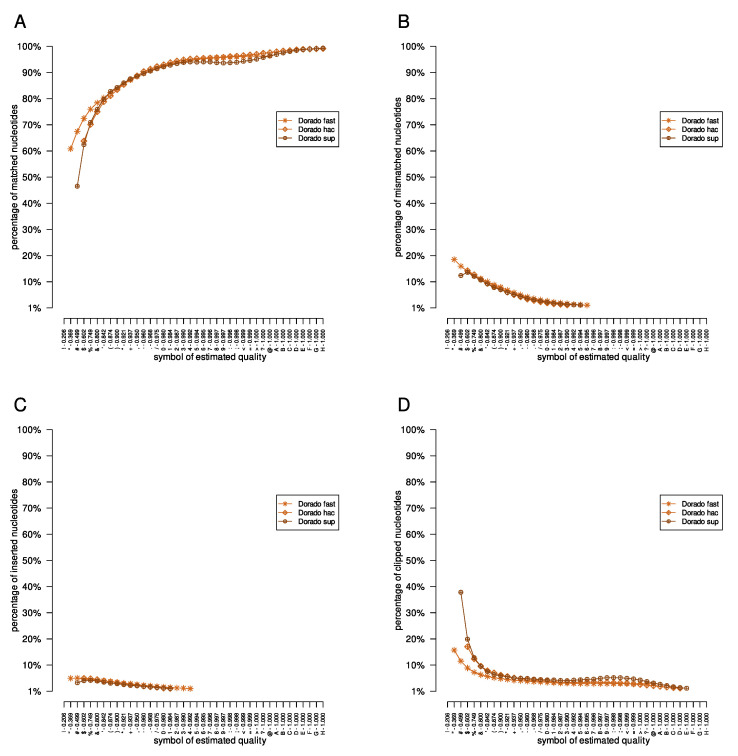
The results of the evaluation of the *Escherichia coli* dataset. The nucleotides classified by the mapping process as matched, mismatched, inserted, and clipped are presented in panels (**A**–**D**), respectively.

Secondly, we examined a different dataset sequenced using Flow Cell R10.4/Kit12/ chemistry. The data used in the study came from [27]; we used three organisms: (I) *Escherichia coli* CFT073 (GenBank accession: NC_004431.1), (II) *Pseudomonas aeruginosa* PAO1 (NC_002516.2), and (III) *Staphylococcus aureus* MRSA252 (NC_002952.2). In the presented experiment, we only used the Dorado basecaller because it is the only one that supports the newest Flow Cell R10.4 (the development of other basecallers examined in this study stopped at Flow Cell 9.4). To explore the effect of the training set on the quality performance of estimation reconstruction, for the Dorado basecaller, we used three trained, default models: dna_r10.4.1_e8.2_400bps_fast@v4.0.0, dna_r10.4.1_e8.2_400bps_hac@v4.0.0, and dna_r10.4.1_e8.2_400bps_sup@v4.0.0. The results are presented in Table 3 and Figure 4, Figure 5 and Figure 6.

Briefly, the results confirm that, as with the previous experiment, the best model for the Dorado basecaller is the dna_r10.4.1_e8.2_400bps_sup@v4.0.0 model (Table 3). Moreover, in Figure 4, it is worth noting that the distribution of quality symbols for the three models differs. It is evident that the best model, i.e., the dna_r10.4.1_e8.2_400bps_sup@v4.0.0 model, has a shifted line toward good quality symbols in relation to the other two models. Analyzing Figure 6A it can be seen that for the Dorado basecaller, the quality symbols reported by all models behaved similarly.

### 3.3. Impact of the Estimated Quality Upon Further Analysis

In the third part of the research presented, we examined the impact of estimated reconstruction quality indicators on the further analysis of genetic data. In particular, we investigated how the estimated quality is used in processes like:

- De novo assembling of long DNA reads (Canu [28], miniasm [29]);
- Resolving ambiguities in the de Bruijn graph (ABySS [30], SPAdes [31]);
- Linking the results of de novo assembling of short reads by long reads (dnaasm-link [32], LINKS [33], SSPACE-LongRead [34]);
- Correcting errors in long reads (Canu [28], LoRDEC [35]);
- Detecting structural variations (Sniffles [36]).

In all of the above-mentioned processes, the impact of the estimated quality of nucleotide reconstruction on the results was examined by comparing the results obtained from three datasets: (I) the raw FASTQ file obtained from the Albacore tool with estimated quality symbols, (II) the FASTQ file with fake quality signs—all quality symbols were set to ‘?’ signs, and (III) the FASTA file—the file obtained from the FASTQ file by removing all lines describing estimated quality indicators. It is worth mentioning that the three datasets mentioned contained identical DNA sequences; they differed only in the estimated quality symbols: (I) real quality symbols from the basecaller, (II) the same symbol of estimated quality equal to ‘?’ for all nucleotides, and (III) no reconstruction quality symbols.

All conducted experiments were performed by launching individual applications with the parameters described in the GitHub repository: https://github.com/wkusmirek/basecalling-quality (accessed on 13 June 2023). The experiments performed, algorithm analysis, and results obtained are described below.

Long DNA Reads De Novo Assembling

First, we checked how the estimated quality of nucleotide reconstruction in the basecalling process affects de novo assembling results. In the study, we used the Canu [28] and miniasm [29] tools. We used three sets (‘FASTQ’, ‘fake FASTQ’, and ‘FASTA’) of DNA reads obtained from *Acinetobacter pittii* reference genome. The results were evaluated by the QUAST [37] tool, the statistics obtained are presented in Table 4. Briefly, the results obtained with the miniasm application are significantly inferior to those obtained with the Canu software (both applications reconstruct a single scaffold, but the quality of the miniasm resultant DNA sequence is poor). However, both the Canu and miniasm tools allowed obtaining identical results for all three analyzed sets of long DNA reads (‘FASTQ’, ‘fake FASTQ’, and ‘FASTA’), which proves that the symbols of the estimated reconstruction quality do not affect the de novo assembling process of long DNA reads by the Canu and miniasm applications.

Resolving Ambiguities in the de Bruijn Graph

Secondly, we examined how the estimated quality of the reconstructed nucleotide can affect the process of resolving ambiguities in the de Bruijn graph [38]. The short DNA reads of the de novo assembling process usually consist of building the de Bruijn graph and generating the resulting DNA sequences from this structure. One of the main challenges in the de novo assembling process is the presence of repetitive DNA fragments leading to the formation of ambiguous fragments in the graph. Each of the ambiguities in the de Bruijn graph results in shorter resultant DNA sequences, the ambiguities could be resolved by mapping long DNA reads to the de Bruijn graph.

In the study, we used two applications supporting hybrid de novo assembling: the ABySS [30] and the SPAdes [31] tools. The experiment was carried out on artificially generated short DNA reads from the *Acinetobacter pittii* reference genome using the pIRS application and three sets of long DNA reads (‘FASTQ’, ‘fake FASTQ’, and ‘FASTA’). The resultant sequences were evaluated by the QUAST tool; the obtained statistics are presented in Table 5. Briefly, the results show that for ABySS and SPAdes, the estimated nucleotide reconstruction quality symbols do not matter in the resolving ambiguities in the de Bruijn graph process; the results for the ‘PET + ONT FASTQ’, ‘PET + ONT fake FASTQ’, and ‘PET + ONT FASTA’ sets are identical. It is also worth noting that for both applications, the usage of long DNA reads improved de novo assembly results in relation to the results obtained only from short DNA reads (rows marked as ‘PET’).

Linking Result of de novo Assembling of Short Reads by Long Reads

Thirdly, long DNA reads can be used in the scaffolding process—to link de novo assembling results of short DNA reads by long DNA reads. To investigate the impact of the estimated quality of nucleotide reconstruction on the results of combining the resulting DNA sequences of the de novo assembly of short DNA reads by long DNA reads, we used dnaasm-link [32], LINKS [33], and SSPACE-LongRead [34] tools. In the experiment, first, we generated a set of short DNA reads from the *Acinetobacter pittii* reference genome by the pIRS [39] read simulator. Then, the short DNA reads were assembled de novo by the ABySS [30] tool, the obtained results were scaffolded with the long DNA reads (‘FASTQ’, ‘fake FASTQ’, and ‘FASTA’) by dnaasm-link, LINKS, and SSPACE-LongRead tools. The final scaffolds were evaluated by the QUAST [37] tool; the results are presented in Table 6. Briefly, all of the tools linked the contigs by the long DNA read the same way, regardless of the presence and values of estimated nucleotide reconstruction quality symbols.

Correcting Errors in Long Reads

Fourthly, we checked the impact of the estimated quality of nucleotide reconstruction in the basecalling process on correcting errors in long DNA reads. Correction of long DNA reads can occur in two ways: (I) correcting by short DNA reads, and (II) correcting by other long DNA reads. In the study presented, we used the LoRDEC [35] and the Canu [28] tools for correcting long reads by the short DNA reads and other long DNA reads, respectively. In the experiment, we corrected three sets (‘FASTQ’, ‘fake FASTQ’, and ‘FASTA’) of DNA reads obtained from the *Acinetobacter pittii* reference genome. The set of short DNA reads was generated from the *Acinetobacter pittii* reference genome by the pIRS [39] reads simulator, and all long DNA reads were evaluated with the BBMap [26] application; the correction results are presented in Table 7. Briefly, both the LoRDEC and Canu applications obtained the same correction results for all three datasets (‘ONT FASTQ’, ‘ONT fake FASTQ’, and ‘ONT FASTA’). The estimated nucleotide reconstruction quality symbols are irrelevant in correcting long DNA reads.

Detecting Structural Variations

Lastly, we checked how the estimated quality determined in the basecalling process affects the number of detected structural variations. In the study, we used the long DNA reads (rel3 release) obtained from the well-characterized NA12787 Genome in a Bottle (GIAB) sample [40,41,42,43]. The experiment carried out involved (I) mapping long DNA reads to the reference genome by the minimap2 [24] tool and (II) detecting the structural variations with the Sniffles [36] application. To speed up the calculations, we limited the research only to chromosome 11; the obtained results are presented in Table 8. Briefly, the number of structural variations detected is the same for all three sets of long DNA reads. Additionally, the detected changes are identical for all three datasets, e.g., the coordinates of the start and end of the structural variation. In the study, we used only chromosome 11, but the conclusions obtained will be the same for other parts of the genome.

## 4. Discussion

In the presented paper, we described the results of the basecaller comparison in terms of the estimated quality of single nucleotide reconstruction (every fourth line in the FASTQ file). The results showed that different basecallers report the estimated quality of nucleotide reconstruction differently, particularly the distributions and ranges of quality symbols. Moreover, basecaller comparisons indicated that the Nanonet tool performed poorly compared to other basecallers. Moreover, the method of estimating the quality of reconstruction proposed in Causalcall and Chiron applications, which calculated the estimated quality as the ratio of the probabilities of the two most probable nucleotides at a given position, does not appropriately present the estimated quality. This can be seen by comparing the analysis of the quality symbols of the mentioned applications with the quality symbols provided by the Albacore, Dorado, Guppy, and Flappie tools (Figure 3).

The presented results open up many paths for further research. Firstly, the applications investigated in the presented study do not use the estimated quality of single nucleotide reconstitution; all reconstructed nucleotides are treated equally, regardless of whether one nucleotide is reconstructed with 50% certainty and the other with 99%. The above observation applies to all estimated qualities of single nucleotide reconstitution, regardless of the basecaller they come from. Consequently, many algorithms could be improved, e.g., in correcting long DNA reads, only nucleotides with a quality symbol denoting low reconstruction probability should be corrected.

Secondly, each of the basecallers presented in the study produced nucleotide reconstruction quality symbols that could represent an entirely different percentage of quality. This observation is evident for low estimated reconstruction quality symbols, where the symbol ‘#’ can mean matching to reference genome rate, e.g., from 60% to 80% depending on the basecaller used. The differences in estimated quality between individual applications can be compensated by recalibrating the provided quality signs. There are several tools for recalibrating NGS data (like ReQON [44] and Lacer [45]). Unfortunately, there is currently no tool to recalibrate the estimated reconstruction quality symbols of the nanopore DNA reads.

Finally, current long DNA read simulators do not provide information about the estimated quality of nucleotide reconstruction in the basecalling process. Adding quality simulations, as with NGS data simulators [46], will enable better development, evaluation, and testing of genetic pipelines dedicated to long nanopore DNA reads.

## 5. Conclusions

As more genomes are sequenced, it will become more desirable to analyze sequencing data, particularly nanopore sequencing data. Here, we compared basecallers in terms of the estimated quality of single nucleotide reconstruction and examined the impact of this estimated quality on further stages of nanopore sequencing data processing. The obtained results show that, currently, the best basecaller that provides DNA reads in the FASTQ format is the Dorado basecaller.

## Figures and Tables

**Table 1:** The table presents the scaling factors for the sum of the estimated reconstruction quality of the nucleotide for the Flappie and Nanonet applications. There are five sets of scaling coefficients for the Nanonet tool, which are selected depending on the type of DNA read and probability of occurrence of the most likely nucleotide (*p*). The estimated reconstruction quality in the Flappie and Nanonet tools are calculated as $-10\cdot (y\cdot \log_{10}\left(1-p\right)+\log_{10}\left(x\right))$.

	x	y
Flappie	1.00000	1.00000
Nanonet template p> 0.1	0.05524	0.70268
Nanonet template p≤ 0.1	0.20938	1.00776
Nanonet complement	0.13120	0.88952
Nanonet 2d	0.02657	0.65590
Nanonet another	1.00000	1.00000

**Table 2:** The statistics of basecalled DNA reads for the *Acinetobacter pittii*, *Haemophilus haemolyticus*, and *Serratia marcescens* datasets. The next columns of the table depict the set of DNA reads (‘Dataset’) and the application (‘Basecaller’) used in the experiment, the number of DNA reads obtained as a result (‘No. of reads’), the sum of the lengths of all DNA reads obtained (‘Sum [Mbp]’), the percentage of all nucleotides that were mapped with the BBMap [26] tool (‘Mapped [%]’) and classified as matched (‘Match [%]’), the substitution error (‘Sub. [%]’), deletion error (‘Del. [%]’), and insertion error (‘Ins. [%]’).

Dataset	Basecaller	No. of Reads	Sum [Mbp]	Mapped [%]	Match [%]	Sub. [%]	Del. [%]	Ins. [%]
*A. pittii*	Albacore	4467	116.63	95.77	86.64	5.35	3.55	4.46
	Causalcall	4467	115.12	92.21	84.36	7.26	4.47	3.91
	Chiron	4467	85.44	81.88	80.43	9.26	5.67	4.64
	Dorado fast	4467	114.94	95.44	90.98	3.66	3.03	2.32
	Dorado hac	4467	114.72	97.50	92.99	2.85	2.67	1.47
	Dorado sup	4467	115.26	97.53	94.09	2.49	2.11	1.30
	Flappie	4467	115.04	95.44	89.66	4.09	3.54	2.71
	Guppy default	4467	115.48	96.47	89.68	4.08	3.22	3.02
	Guppy kp	4467	113.84	96.35	87.60	4.50	4.74	3.16
	Guppy kp-big-net	4467	114.99	97.32	89.73	3.83	3.62	2.82
	Nanonet	7702	118.18	67.33	84.05	6.05	6.72	3.18
*H. haemolyticus*	Albacore	8669	88.15	94.28	86.83	4.64	2.79	5.74
	Causalcall	8669	52.51	2.24	78.03	10.73	5.46	5.78
	Chiron	8669	77.06	43.05	78.28	10.78	5.11	5.83
	Dorado fast	8669	84.88	58.04	89.64	4.01	2.77	3.57
	Dorado hac	8669	71.81	75.65	91.09	3.62	3.03	2.24
	Dorado sup	8669	91.33	59.77	93.43	2.58	2.04	1.95
	Flappie	8669	77.33	77.34	88.42	4.35	3.96	3.27
	Guppy default	8669	86.29	95.09	88.62	4.21	3.13	4.04
	Guppy kp	8669	84.81	95.67	87.84	4.56	4.07	3.53
	Guppy kp-big-net	8669	86.31	97.50	90.14	3.47	2.80	3.59
	Nanonet	15,956	90.95	65.90	86.19	5.02	4.85	3.94
*S. marcescens*	Albacore	16,742	141.04	88.96	85.36	5.75	4.18	4.71
	Causalcall	16,742	140.10	84.93	83.73	7.24	4.68	4.35
	Chiron	16,742	130.65	84.50	82.88	6.98	5.10	5.04
	Dorado fast	16,742	139.75	87.82	89.23	4.32	3.41	3.04
	Dorado hac	16,742	139.16	91.26	91.67	3.29	3.10	1.93
	Dorado sup	16,742	139.60	91.65	92.87	2.92	2.56	1.65
	Flappie	16,742	139.81	88.07	87.77	4.81	4.02	3.40
	Guppy default	16,742	140.32	89.46	87.22	5.07	4.01	3.70
	Guppy kp	16,742	138.09	90.29	86.69	4.91	5.04	3.36
	Guppy kp-big-net	16,742	138.98	91.24	88.83	3.99	4.19	2.99
	Nanonet	34,046	151.49	45.88	81.27	7.24	7.01	4.48

**Table 3:** The statistics of basecalled DNA reads for the *Escherichia coli*, *Pseudomonas aeruginosa*, and *Staphylococcus aureus* datasets. The next columns in the table depict the set of DNA reads (‘Dataset’) and the application (‘Basecaller’) used in the experiment, the number of DNA reads obtained as a result (‘No. of reads’), the sum of the lengths of all DNA reads obtained (‘Sum [Mbp]’), the percentage of all nucleotides that were mapped with the BBMap [26] tool (‘Mapped [%]’) and classified as matched (‘Match [%]’), the substitution error (‘Sub. [%]’), deletion error (‘Del. [%]’), and insertion error (‘Ins. [%]’).

Dataset	Basecaller	No. of Reads	Sum [Mbp]	Mapped [%]	Match [%]	Sub. [%]	Del. [%]	Ins. [%]
*E. coli*	Dorado fast	3994	10.35	87.32	86.70	6.27	4.32	2.69
	Dorado hac	3994	10.55	92.31	93.02	3.07	2.19	1.72
	Dorado sup	3994	10.68	93.44	94.27	2.49	1.83	1.40
*P. aeruginosa*	Dorado fast	3994	19.25	89.53	89.97	4.57	3.13	2.33
	Dorado hac	3994	19.42	90.51	95.81	1.79	1.34	1.06
	Dorado sup	3994	19.49	91.06	96.94	1.30	1.00	0.77
*S. aureus*	Dorado fast	4000	11.70	95.56	90.08	4.59	2.54	2.77
	Dorado hac	4000	11.72	97.36	95.82	1.81	1.00	1.35
	Dorado sup	4000	11.69	97.53	96.99	1.26	0.77	0.97

**Table 4:** The statistics of the Canu and miniasm de novo assembly results. The following columns of the table depict the set of DNA reads (‘Dataset’), the number of DNA scaffolds obtained as a result (‘No. of scaff.’), the sum of the lengths of all DNA scaffolds obtained (‘Sum [Mbp]’), the size of the most extensive alignment of the resultant scaffold to the reference genome (‘Largest align. [bp]’), the N50 statistic calculated based on aligned blocks instead of contigs (‘NA50 scaff. [bp]’), the number of errors in the resultant scaffolds per 100 kbp divided into two groups (‘Errors per 100 kbp’): mismatch errors (‘mismatch’) and indel errors (‘indel’).

						Errors per 100 kbp	
	Dataset	No. of Scaff.	Sum [bp]	Largest Align. [bp]	NA50 scaff. [bp]	Mismatch	Indel
Canu	ONT FASTQ	1	3,840,618	3,839,076	3,839,076	24.23	521.99
	ONT fake FASTQ	1	3,840,618	3,839,076	3,839,076	24.23	521.99
	ONT FASTA	1	3,840,618	3,839,076	3,839,076	24.23	521.99
miniasm	ONT FASTQ	1	3,837,407	773	-	1346.15	2500.00
	ONT fake FASTQ	1	3,837,407	773	-	1346.15	2500.00
	ONT FASTA	1	3,837,407	773	-	1346.15	2500.00

**Table 5:** Statistics of hybrid de novo assembling with ABySS and SPAdes applications. The rows with the dataset marked as ‘PET’ show the results of de novo assembling the short DNA reads, without using long nanopore reads. The next columns of the table depict the set of DNA reads (‘Dataset’), the number of DNA scaffolds obtained as a result (‘No. of scaff.’), the sum of the lengths of all DNA scaffolds obtained (‘Sum [Mbp]’), the size of the most extensive alignment of the resultant scaffold to the reference genome (‘Largest align. [bp]’), the N50 statistic calculated based on aligned blocks instead of contigs (‘NA50 scaff. [bp]’), the number of errors in the resultant scaffolds per 100 kbp divided into two groups (‘Errors per 100 kbp’): mismatch errors (‘mismatch’) and indel errors (‘indel’).

						Errors per 100 kbp	
	Dataset	No. of Scaff.	Sum [bp]	Largest Align. [bp]	NA50 Scaff. [bp]	Mismatch	Indel
ABySS	PET	103	3,841,385	267,798	84,121	3.51	0.76
	PET + ONT FASTQ	86	3,827,061	318,468	110,838	3.51	0.76
	PET + ONT fake FASTQ	86	3,827,061	318,468	110,838	3.51	0.76
	PET + ONT FASTA	86	3,827,061	318,468	110,838	3.51	0.76
SPAdes	PET	110	3,740,469	314,157	87,580	0.75	0.59
	PET + ONT FASTQ	2	3,814,351	2,629,607	2,629,607	1.15	0.50
	PET + ONT fake FASTQ	2	3,814,351	2629607	2,629,607	1.15	0.50
	PET + ONT FASTA	2	3,814,351	2,629,607	2,629,607	1.15	0.50

**Table 6:** The table presents statistics of linking contigs (DNA sequences de novo assembled from short DNA reads by the ABySS tool) by the long DNA reads (‘ONT FASTQ’, ‘ONT fake FASTQ’, ‘ONT FASTA’) with dnaasm-link, LINKS, and SSPACE-LongRead tools. The following columns of the table depict the set of DNA reads (‘Dataset’), the number of DNA scaffolds obtained as a result (‘No. of scaff.’), the sum of the lengths of all DNA scaffolds obtained (‘Sum [Mbp]’), the size of the most extensive alignment of the resultant scaffold to the reference genome (‘Largest align. [bp]’), the N50 statistic calculated based on the aligned blocks instead of contigs (‘NA50 scaff. [bp]’), the number of errors in the resultant scaffolds per 100 kbp divided into two groups (‘Errors per 100 kbp’): mismatch errors (‘mismatch’) and indel errors (‘indel’).

						Errors per 100 kbp	
	Dataset	No. of Scaff.	Sum [bp]	Largest Align. [bp]	NA50 Scaff. [bp]	Mismatch	Indel
	contigs	103	3,841,385	267,798	84,121	3.51	0.76
dnaasm-link	ONT FASTQ	55	3,857,847	404,444	256,914	3.53	1.34
	ONT fake FASTQ	55	3,857,847	404,444	256,914	3.53	1.34
	ONT FASTA	55	3,857,847	404,444	256,914	3.53	1.34
LINKS	ONT FASTQ	94	3,842,749	318,434	99,326	3.51	0.92
	ONT fake FASTQ	94	3,842,749	318,434	99,326	3.51	0.92
	ONT FASTA	94	3,842,749	318,434	99,326	3.51	0.92
SSPACE-LongRead	ONT FASTQ	38	3,937,981	702,998	423,517	3.53	0.79
	ONT fake FASTQ	38	3,937,981	702,998	423,517	3.53	0.79
	ONT FASTA	38	3,937,981	702,998	423,517	3.53	0.79

**Table 7:** The long DNA reads of correction statistics with short DNA reads (LoRDEC) and long DNA reads (Canu). The ‘ONT raw’ row presents statistics of the raw, uncorrected long DNA reads corrected later by LoRDEC and Canu tools. The following columns of the table depict the correcting tool (‘Application’), the set of DNA reads (‘Dataset’), the number of DNA reads obtained as a result (‘No. of reads’), the sum of the lengths of all DNA reads obtained (‘Sum [Mbp]’), the percentage of all nucleotides that were mapped with the BBMap [26] tool (‘Mapped [%]’), and classified as matched (‘Match [%]’), substitution error (‘Sub. [%]’), deletion error (‘Del. [%]’), and insertion error (‘Ins. [%]’).

Application	Dataset	No. of Reads	Sum [Mbp]	Mapped [%]	Match [%]	Sub. [%]	Del. [%]	Ins. [%]
	ONT raw	4467	116.63	95.77	86.64	5.35	3.55	4.46
LoRDEC	ONT FASTQ	4467	115.42	96.99	98.72	0.64	0.42	0.22
	ONT fake FASTQ	4467	115.42	96.99	98.72	0.64	0.42	0.22
	ONT FASTA	4467	115.42	96.99	98.72	0.64	0.42	0.22
Canu	ONT FASTQ	4328	110.52	99.92	98.12	0.23	1.47	0.18
	ONT fake FASTQ	4328	110.52	99.92	98.12	0.23	1.47	0.18
	ONT FASTA	4328	110.52	99.92	98.12	0.23	1.47	0.18

**Table 8:** The table presents the number of structural variations detected in chromosome 11 of the NA12787 sample. The variations were divided into five groups: deletions (‘Del’), insertions (‘Ins.’), inversions (‘Inv.’), duplications (‘Dup.’), and translocations (‘Trans.’). The experiment was carried out on three sets of the same long DNA reads: (I) raw sets of reads with real symbols of estimated quality (‘ONT FASTQ’), (II) DNA reads for which all quality symbols were changed to the ‘?’ sign (‘ONT fake FASTQ’), and (III) DNA reads without symbols, denoting the estimated quality of the nucleotide reconstruction (‘ONT FASTA’).

Dataset	Del.	Ins.	Inv.	Dup.	Trans.
ONT FASTQ	1645	368	7	7	0
ONT fake FASTQ	1645	368	7	7	0
ONT FASTA	1645	368	7	7	0

## Data Availability

The benchmarking procedure, as well as test data, are publicly accessible at the GitHub repository: https://github.com/wkusmirek/basecalling-quality (accessed on 13 June 2023). The Docker image is available at: https://hub.docker.com/r/wkusmirek/basecalling-quality (accessed on 13 June 2023).

## Supplementary Materials

The following are available at https://www.mdpi.com/article/10.3390/s23156787/s1.
