# Supplementary material for: Estimated Nucleotide Reconstruction Quality Symbols of Basecalling Tools for Oxford Nanopore Sequencing
Source: Sensors (Basel). 2023 Jul 29;23(15):6787. doi: 10.3390/s23156787 (PMC10422362; doi:10.3390/s23156787)
Supplement: Supplementary file 1 [file sensors-23-06787-s001.zip › sensors-2504358-supplementary.pdf]

Estimated nucleotide reconstruction quality symbols of basecalling tools for Oxford Nanopore sequencing

Wiktor Kuśmirek

Supplementary Materials

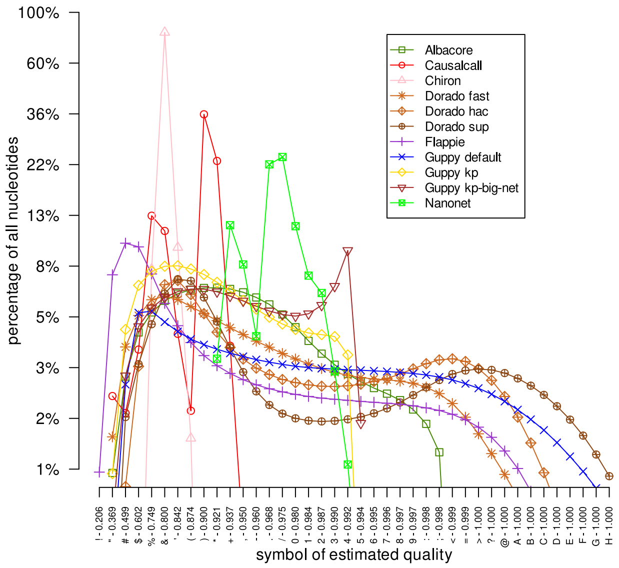

Figure S1: Reconstructed DNA reads quality symbol distribution for *Haemophilus haemolyticus* dataset.

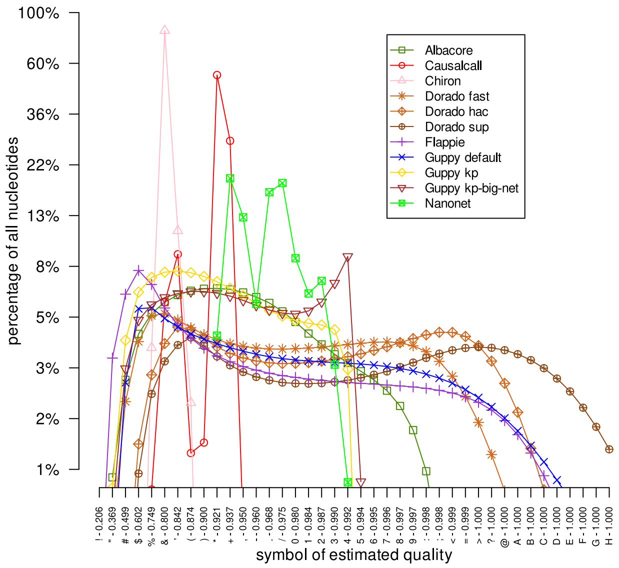

Figure S2: Reconstructed DNA reads quality symbol distribution for *Serratia marcescens* dataset.

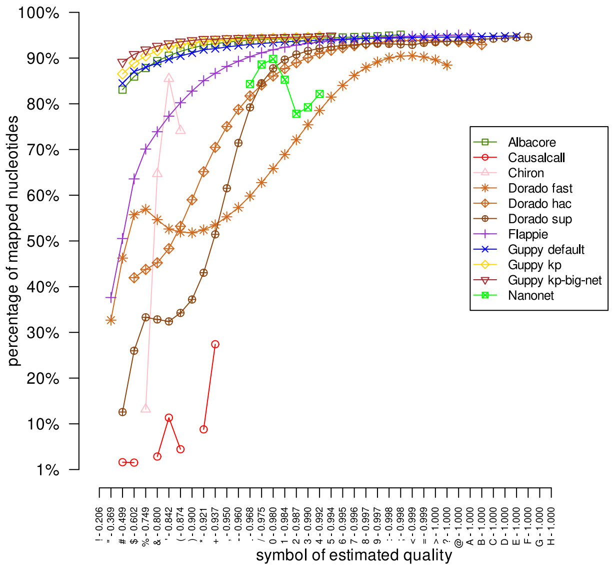

Figure S3: Number of nucleotides mapped by the minimap2 tool for *Haemophilus haemolyticus* dataset.

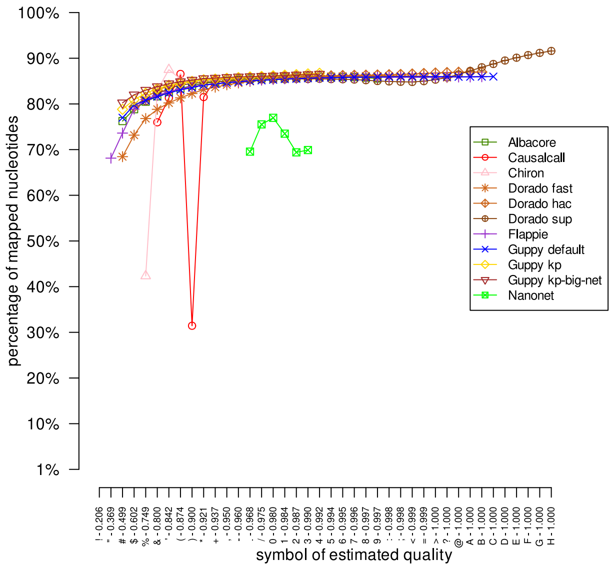

Figure S4: Number of nucleotides mapped by the minimap2 tool for *Serratia marcescens* dataset.

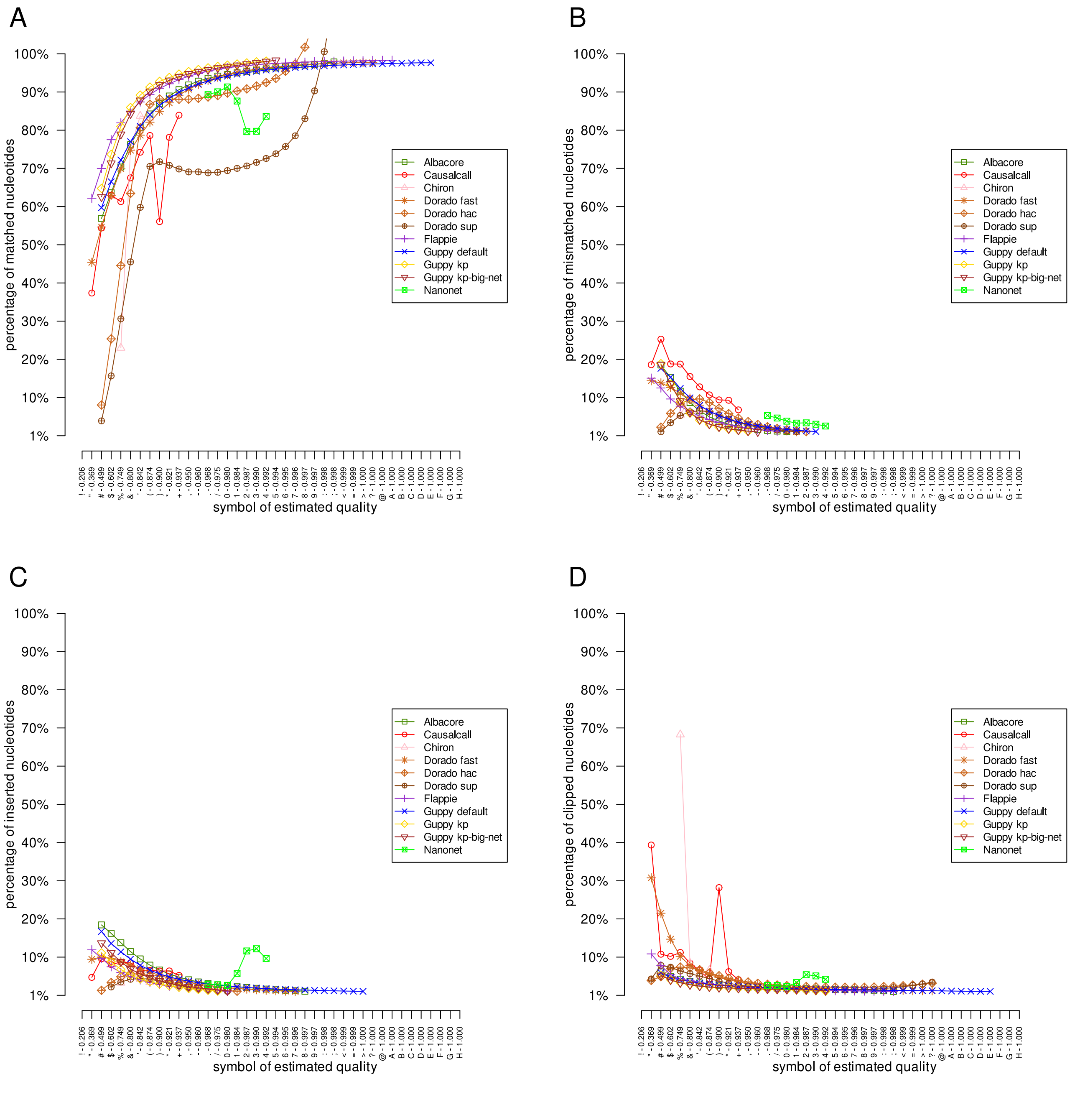

Figure S5: The results of the evaluation of the *Haemophilus haemolyticus* dataset.

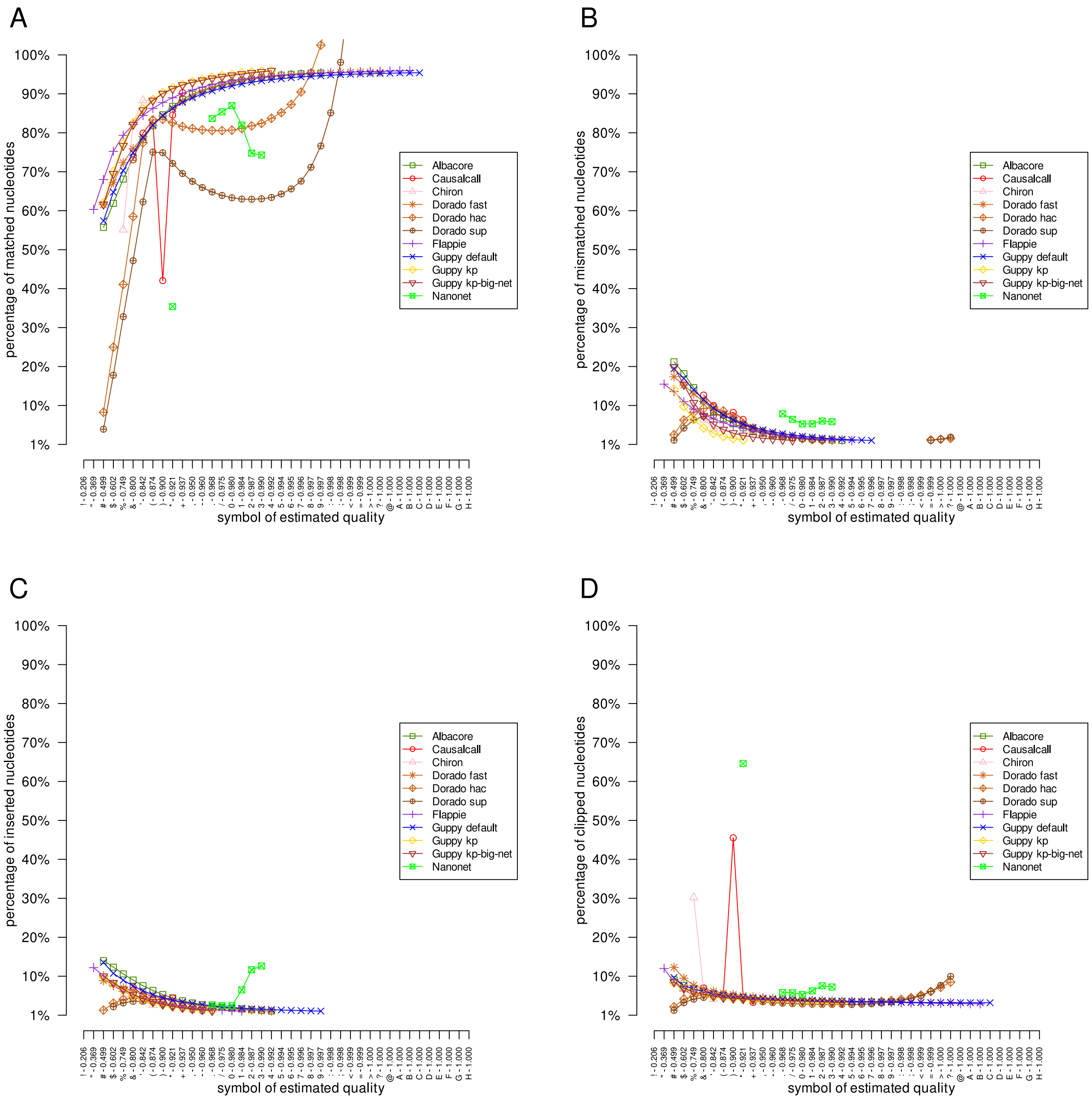

Figure S6: The results of the evaluation of the *Serratia marcescens* dataset.

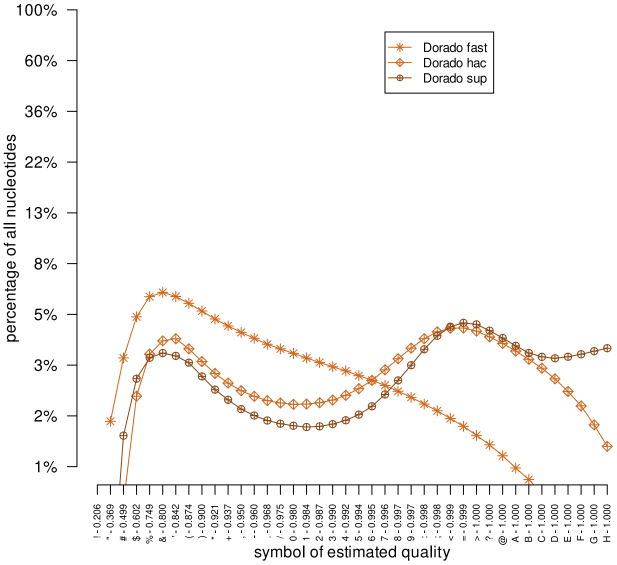

Figure S7: Reconstructed DNA reads quality symbol distribution for *Pseudomonas aeruginosa* dataset.

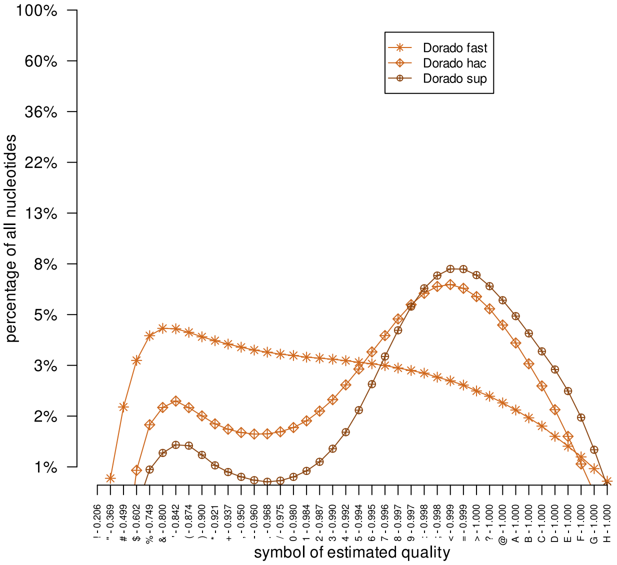

Figure S8: Reconstructed DNA reads quality symbol distribution for *Staphylococcus aureus* dataset.

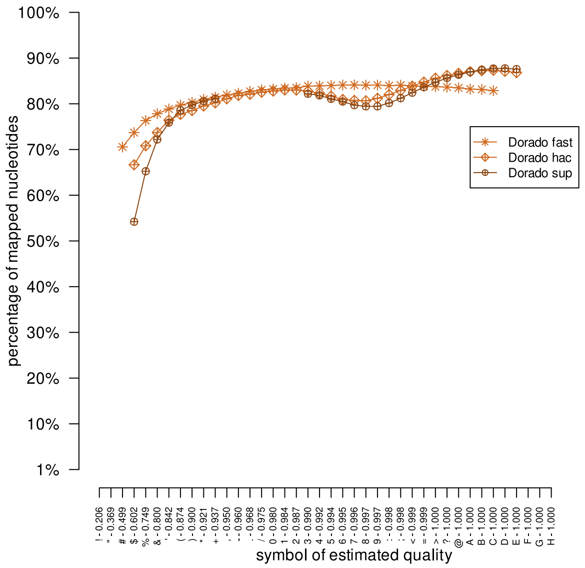

Figure S9: Number of nucleotides mapped by the minimap2 tool for *Pseudomonas aeruginosa* dataset.

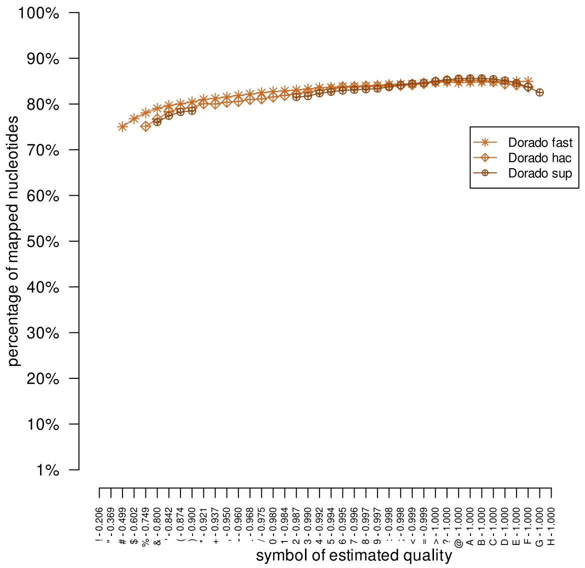

Figure S10: Number of nucleotides mapped by the minimap2 tool for *Staphylococcus aureus* dataset.

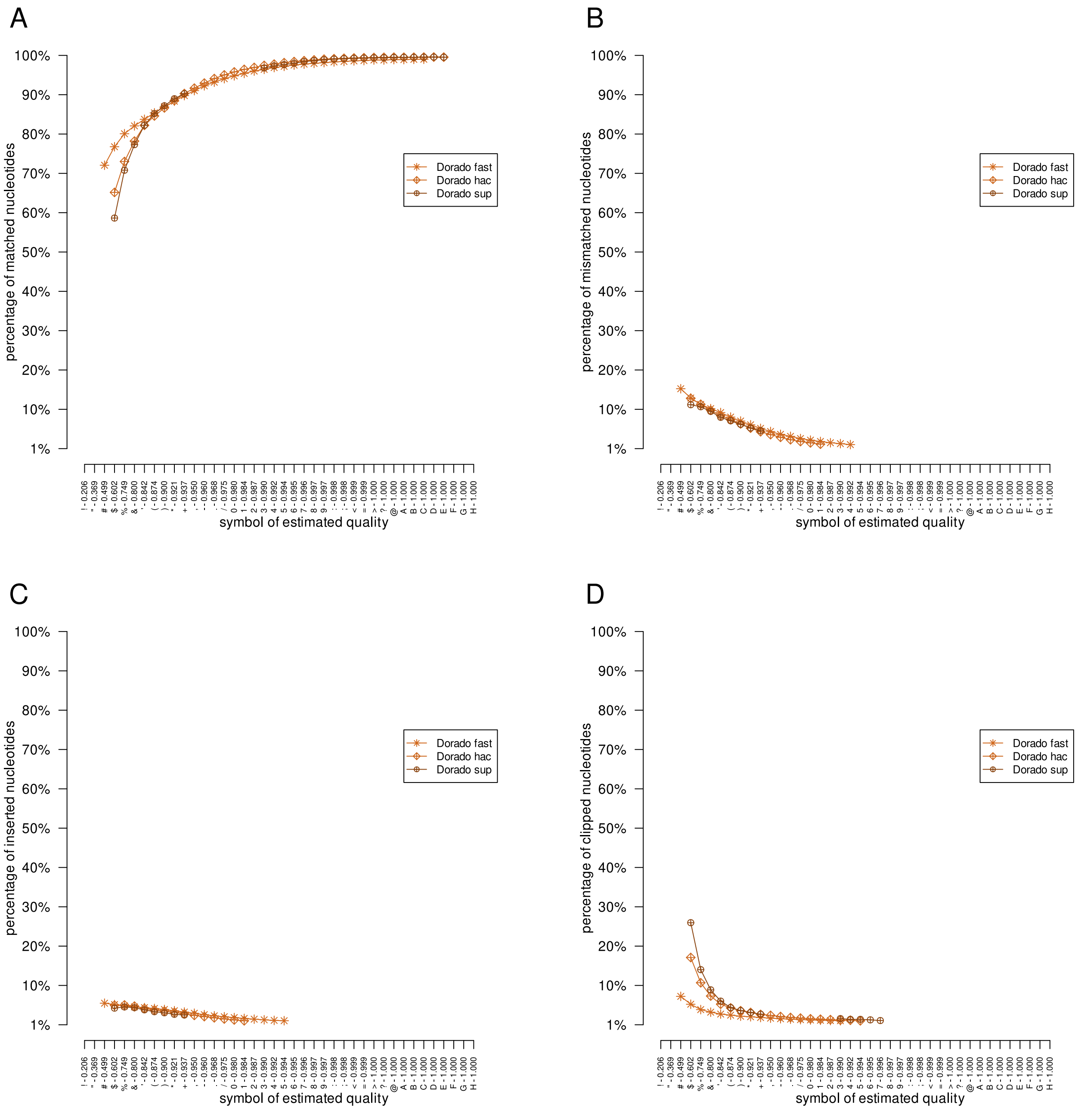

Figure S11: The results of the evaluation of the *Pseudomonas aeruginosa* dataset.

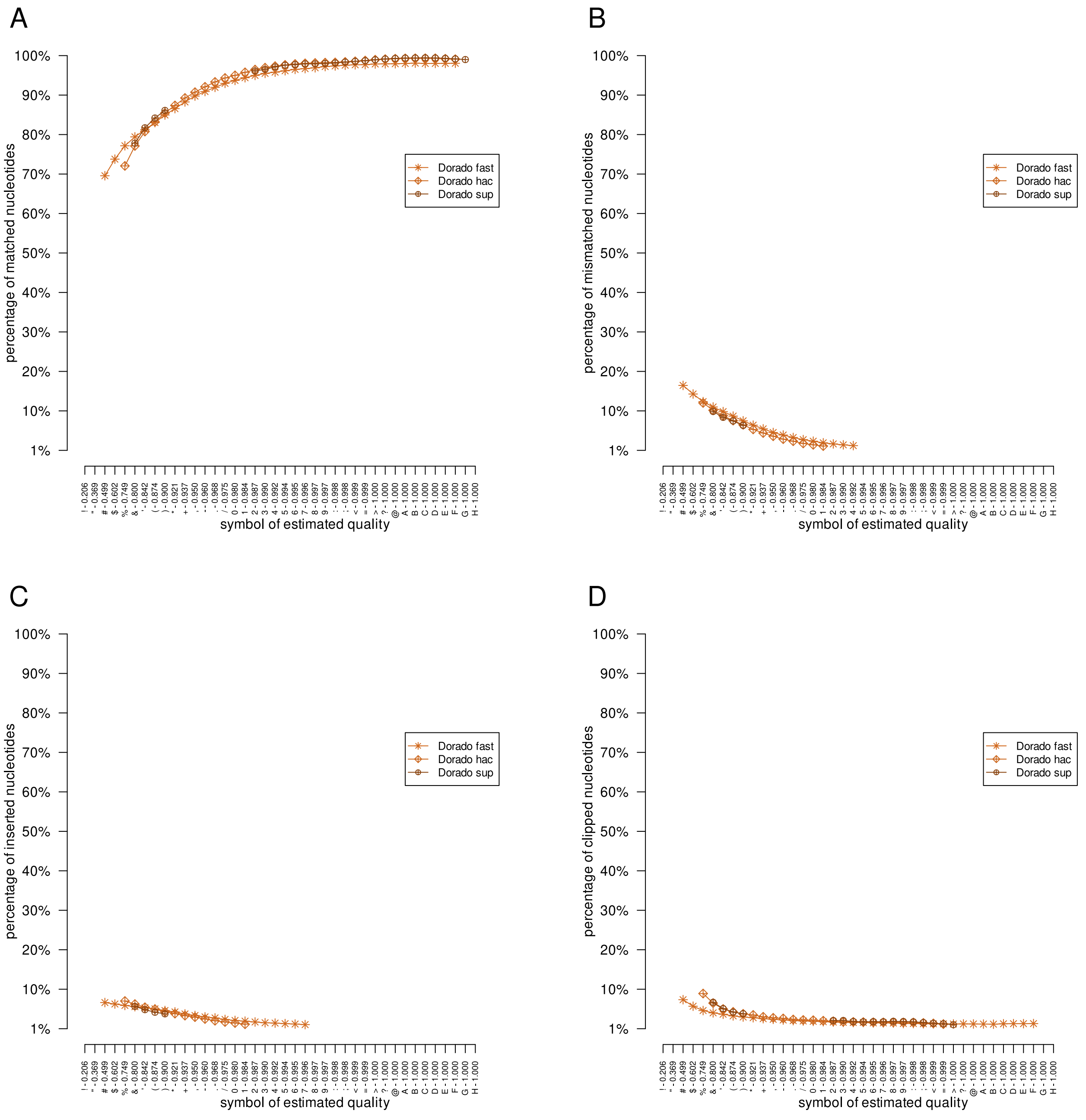

Figure S12: The results of the evaluation of the *Staphylococcus aureus* dataset.
